# Effect of two alternative methods of pooling sputum prior to testing for tuberculosis with genexpert MTB/RIF

**DOI:** 10.1186/s12879-019-3778-9

**Published:** 2019-04-27

**Authors:** Nguyen Thi Bich Phuong, Nguyen Thu Anh, Nguyen Van Son, Vitali Sintchenko, Jennifer Ho, Greg J. Fox, Nguyen Viet Nhung, Guy B. Marks

**Affiliations:** 1 Woolcock Institute of Medical Research, 298 Kim Ma street, Ba Dinh district, Ha Noi, Vietnam; 2 Faculty of Medicine and Health, The University of Sydney, Sydney, Australia; 3 Centre for Social Disease Control, Ca Mau, Vietnam; 4 National TB Control Program, Hanoi, Vietnam; 5 South Western Sydney Clinical School, University of NSW, Sydney, Australia

**Keywords:** Nucleic acid amplification, Diagnostic test characteristics, Sensitivity

## Abstract

**Background:**

Pooling sputum specimens is one potential strategy for reducing the cost of using Xpert MTB/RIF, a rapid polymerase chain reaction (PCR)-based test, for the diagnosis of pulmonary tuberculosis. We sought to compare the sensitivity of two alternative method of pooling.

**Methods:**

Patients referred for assessment for TB, whose initial sputum was Xpert MTB positive, were recruited and their sputum specimens were pooled for analysis with sputum specimens that were Xpert MTB negative. Two alternative pooling strategies were employed: one in which the concentration of sample reagent (buffer) was maintained at 2:1 (standard), in accordance with the manufacturer’s instructions, and another in which the concentration of sample reagent was reduced to 1:1.

**Results:**

We tested 101 Xpert MTB positive sputum specimens. Among these, 96% of valid test results (95% confidence interval (CI) 89–99%) were positive using the “standard buffer method”. Using the “reduced buffer pooling” method 94% of valid test results (95% CI 87–98%) were positive. McNemar’s test for the difference in paired proportions did not reach statistical significance (*P* = 0.56).

**Conclusion:**

We have confirmed that pooling of two sputum specimens for testing in a single cartridge is a valid method of reducing the number of cartridges required when using Xpert MTB to detect pulmonary tuberculosis. Two alternative pooling strategies tested here yielded similar results.

**Trial registration:**

The present study was conducted within the Active Casefinding in Tuberculosis (ACT3) Trial. The ACT3 Trial had been registered with Australian and New Zealand Clinical Trials Register on 8th April, 2014. The trial registration number is ACTRN12614000372684. (Retrospectively registered).

## Background

The increasing recognition that active case finding has a role to play as a component of strategies to accelerate progress towards tuberculosis (TB) elimination highlights the need to optimise screening strategies [1–4]. Nucleic acid amplification tests, such as Xpert MTB/RIF (Cepheid, Sunnyvale, CA, USA), have good test performance characteristics, which make them attractive in this context [5, 6]. However, their unit cost remains a barrier to implementation at scale, especially in countries with a high incidence of tuberculosis.

Zishiri and colleagues have shown, in 20 acid-fast bacilli smear positive and 17 smear negative sputum specimens that were *Mycobacterium tuberculosis* (MTB) culture positive and Xpert MTB positive, that pooling up to five-fold (that is, each with four negative sputum samples collected from other individuals) was associated with acceptable sensitivity when implemented in a reference laboratory [7]. This pooling ratio was based on modelling that identified a 1-in-5 ratio as optimal when the expected prevalence of positive tests was 3%. In a larger study, Abdurrahman and colleagues subsequently showed that pooling up to four fold also resulted in acceptable sensitivity for diagnostic sputum specimens [8]. These findings imply that, for screening TB suspects, pooling up to four- or five-fold is feasible and retains good sensitivity.

In some screening settings, such as general community-wide screening, the prior probability of TB will be lower and likely concentration of *M. tuberculosis* in sputum specimens submitted for testing will be lower. Hence, it is encouraging that we have shown, using sputum specimens spiked with a low concentration of *M. tuberculosis* and diluted from 2- to 12-fold that, despite an expected linear increase in cycle threshold with increasing dilution, the majority of diluted specimens were still Xpert MTB positive [9]. However, in order to maintain optimal sensitivity, it seems plausible that a pooling strategy that retains the original concentration of *M. tuberculosis* organisms would be preferred.

The goal of the present study was to establish whether reducing the ratio of buffer used in the preparation of sputum for testing, in order to retain a higher concentration of sputum in the test solution, would yield a higher level of test sensitivity when sputum specimens are pooled prior to Xpert MTB/RIF analysis. Hence, we have compared two strategies for pooling sputum specimens in a ratio of 1:2 prior to Xpert testing: one in which the sputum concentration is halved (“standard buffer pooling”) and another, in which volume of buffer is reduced to maintain the original sputum concentration (“reduced buffer pooling”). The objective of this study was to compare the sensitivity of these two methods.

## Methods

A cross-sectional study was conducted in Xpert MTB positive sputum specimens collected from a consecutive series of consenting patients aged ≥18 years who were being evaluated for TB at the Centre for Social Disease Prevention (CSDP), in Ca Mau city, Ca Mau province, Vietnam. These were patients who presented to, or were referred to, the CSDP because of a suspicion of TB. The project was approved by the Human Research Ethics Committee of the University of Sydney (2016/454). Evaluated patients who consented to participate were asked to provide a one spot sputum of at least 2 mL in volume. This sputum specimen was mixed with an equal volume of the supplied sample reagent (Cepheid, Sunnyvale, CA, USA) and vortexed to ensure homogenization. If the solution remained viscous, it was allowed to stand for 30 min to allow further liquefaction. This solution was used as a “stock solution” in subsequent steps. All the processing and pooling steps were performed in a Class I Biological Safety Cabinet (BSC) BYKG-I (Biobase, Jinan, China).

First, we emulated the standard procedure recommended by the manufacturer. A 2 mL aliquot of the stock solution was mixed with a further 1 mL of the sample reagent, to yield a final sputum:buffer concentration of 1:2. This solution was tested in an Xpert MTB/RIF cartridge in accordance with the manufacturer’s instructions. If this standard Xpert test was positive (for MTB), we proceeded to test pooled specimens. After this step, each stock solution was labelled “Xpert positive stock solution” or “Xpert negative stock solution” depending on the result of this initial, standard Xpert MTB test.

Each sample that produced a Xpert positive stock solution was used to test two pooling procedures. In the “standard buffer pooling” method, 1 mL of the Xpert positive stock solution was diluted with 0.5 mL of sample reagent and 1 mL of Xpert negative stock solution with a further 0.5 mL of sample reagent added. Finally, this 3 mL solution included 2 mL of sample reagent and 1 mL of sputum (0.5 mL from each of two, pooled, specimens). Hence, the sputum:buffer ratio was 1:2. An aliquot of 2 mL was tested in an Xpert MTB/RIF cartridge.

In the “reduced buffer pooling” method, a 1.5 mL aliquot of the Xpert positive stock solution was mixed with a 1.5 mL aliquot of the Xpert negative stock solution, without addition of further sample reagent. Hence, the sputum:buffer ratio was 1:1. An aliquot of 2 mL was tested in an Xpert MTB/RIF cartridge.

The cycle threshold (C_T_) for Probe B was taken as the final result of the test unless this was missing or not detected, in which case the lowest C_T_ value of the other probes was used. Results were reported using the manufacturer’s recommended semi-quantitative classification of Ct values. Ct values were categorized as very low (> 28 cycles), low (23–28 cycles), medium (16–22 cycles) and high (< 16 cycles) [10].

The sensitivity of each method was calculated as proportion of pooled samples that were positive. The difference between the two methods was tested using McNemar’s test. We sought to test 80 samples using both methods, giving 80% power to detect a difference between the two methods in the proportion of discordant results of 9.8%, if the overall proportion of discordant pairs was 10, and 14.2%, if the overall proportion of discordant pairs was 20%.

## Results

We performed Xpert MTB testing, using the standard method with undiluted sputum samples collected from 262 consenting patients being evaluated for TB who produced ≥2 mL sputum for testing. Among these, 101 (38.6%) patients produced samples that were Xpert MTB positive (Table 1). The numbers of Xpert MTB positive specimens classified as high, medium, low and very low on Xpert testing were 13 (12.9%), 47 (46.5%), 28 (27.7%), and 13 (12.9%), respectively. Using the “standard buffer pooling” method there were two “error” readings. Of the remaining samples, 95 (96, 95% confidence interval (CI) 89–99%) tested positive on Xpert. Using the “reduced buffer pooling” method there were eight “error” readings and a further four specimens were not tested for technical reasons. Of the remaining specimens, 84 (94, 95% confidence interval 87 to 98%) tested positive. McNemar’s test for the difference in paired proportions was not significant for the number of positive test results (*P* = 0.56), but the number error test results was significantly higher (*P* = 0.03).

**Table 1:** Xpert MTB/RIF results by method of pooling

		Standard Buffer Pooling Method			
		MTB detected	MTB not detected	Error	Total
Reduced Buffer Pooling Method	MTB detected	82	1	1	84
	MTB not detected	2	3	0	5
	Error	7	0	1	8
	Not tested	4	0	0	4
	Total	95	4	2	101

*MTB* Mycobacterium tuberculosis

Three specimens were negative using both pooling techniques. All three of these had C_T_ values for the original unpooled specimen > 30. Three other specimens tested negative with one pooling technique, but were detected by the other. These specimens had C_T_ values in the original unpooled specimen of 27.7, 28.3 and 28.7, respectively.

## Discussion

We compared two method of preparing pooled sputum specimens for testing using Xpert MTB/RIF and have shown that both methods had acceptable sensitivity for testing Xpert MTB positive sputum collected from people being investigated for TB. The reduced buffer pooling method was not more sensitive than the standard buffer pooling method. The sensitivity of the standard pooling method was very high. Four false negative tests were observed, meaning it was very difficult to demonstrate that the reduced buffer pooling method had superior sensitivity. It remains uncertain whether, with lower concentrations of organisms present, as for example in the screening context [6, 9], the standard buffer pooling technique may have lower sensitivity. We did find an increase in “error” results in specimens prepared using the reduced buffer pooling technique, compared to the standard buffer pooling technique. This may have been attributable to the increased viscosity of the specimens. While “error” results will lead to an increased cost, due to the need to re-collect and/or re-test specimens with an additional cartridge, this problem did not threaten our ability to obtain a valid test result, because a non-diagnostic result always lead to re-testing.

The use of a lower concentration of buffer in the preparation of the specimen for testing may lead to biosafety concerns, since the safety of the current methodology, in which a BSC is not recommended, is premised on the standard concentration of buffer being used [11]. This requires further evaluation but, for this study, we elected to perform the procedure in a Class I BSC. This may be feasible in the setting of active case finding, where the procedure is performed in a central laboratory, but is unlikely to be feasible in peripheral laboratories where such facilities are unavailable.

The main limitation of this study was that the sensitivity of the standard buffer pooling technique was higher than expected, meaning that it did not have sufficient power to test whether the reduced buffer pooling technique was more sensitive. A further limitation of this study is that the participants were all being evaluated for a clinically suspected diagnosis of TB and hence, had a relatively high pre-test probability and, probably, a relative high concentration of mycobacterial DNA in their sputum. This might not be relevant to other contexts, such as active case finding, in which the prevalence and concentration of *M. tuberculosis* may be lower.

## Conclusion

The findings support previous work [7, 9] that pooling of sputum specimens is a feasible strategy for reducing the cost of Xpert testing for the diagnosis of pulmonary tuberculosis. Both standard and reduced buffer methods would appear to be effective for this purpose. The role of this procedure in active case finding requires further evaluation.

## Abbreviations

BSC: Bio-Safety Cabinet
CSDP: Centre for Social Disease Prevention
C_T_: Cycle threshold
DNA: Deoxyribose Nucleic Acid
MTB: *Mycobacterium tuberculosis*
RIF: Rifampicin
TB: Tuberculosis
USA: United State of Americal
